# Recurrent Diplopia in a Pediatric Patient with Bickerstaff Brainstem Encephalitis

**DOI:** 10.1155/2016/5240274

**Published:** 2016-05-17

**Authors:** Scott A. McLeod, Wallace Wee, Francois D. Jacob, Isabelle Chapados, Francois V. Bolduc

**Affiliations:** ^1^Section of Developmental Pediatrics, Alberta Children's Hospital, 2888 Shaganappi Trail NW, Calgary, AB, Canada T3B 6A8; ^2^Department of Pediatrics, Edmonton Clinic Health Academy, University of Alberta, 11405 87 Avenue, Edmonton, AB, Canada T6G 1C9

## Abstract

*Introduction*. Acute complete external ophthalmoplegia is a rare finding in clinical practice that is associated with diseases affecting the neuromuscular junction, the oculomotor nerves, or the brainstem. Ophthalmoplegia has been reported with acute ataxia in Miller Fisher syndrome (MFS) and Bickerstaff brainstem encephalitis (BBE). Up to 95% of these cases are associated with anti-GQ1b antibodies. Only a small number of cases of anti-GQ1b negative MFS have been documented in pediatric patients. This is the first case reporting a recurrence of ocular symptoms in an anti-GQ1b antibody negative patient with BBE.* Case Presentation*. An 8-year-old Caucasian boy presented with complete external ophthalmoplegia without ptosis, cerebellar ataxia, and a disturbance of consciousness. He had recently recovered from a confirmed* Campylobacter jejuni* infection. On subsequent laboratory testing he was anti-GQ1b antibody negative. He had a recurrence of diplopia at four-week follow-up.* Conclusions*. This patient's recurrence of diplopia was treated with a five-week course of oral corticosteroids which did not worsen his condition, and this may be a therapeutic option for similar patients. We will discuss the symptoms and treatment of reported pediatric cases of anti-GQ1b antibody negative cases of MFS and the variation between cases representing a spectrum of illness.

## 1. Introduction

Bickerstaff brainstem encephalitis (BBE) is characterized by external ophthalmoplegia, ataxia, and a disturbance of consciousness [1–3]. Most commonly, this syndrome is associated with anti-GQ1b antibodies [4–6]. As further information is known about the immune mechanisms of this illness, it is thought to be on a spectrum of illness with Guillain-Barré syndrome and MFS [3, 5, 6]. There have been rare episodes described of negative anti-GQ1b antibody cases of MFS, with no recurrences of symptoms reported. In this report, we describe the first anti-GQ1b antibody negative case with recurrent symptoms. We discuss the difficulty in making a formal diagnosis in patients who present on the spectrum of illness between MFS and BBE and the most current literature around treatment of anti-GQ1b antibody negative cases.

## 2. Case Presentation

This 8-year-old, previously healthy, right-handed boy presented to the emergency department with new onset horizontal binocular diplopia in all directions of gaze, headache, ataxia, and decreased oral intake. No ptosis or eyelid involvement was initially noted. He had antecedent emesis and diarrhea commencing ten days before for one-week duration, although these symptoms had resolved prior to presentation. Prior to presentation he had increasing headache frequency and had an episode of decreased level of consciousness; he fell from a couch, became stiff, and reportedly made incomprehensible sounds for 5–10 seconds. He had a prior six-month history of weekly headaches located in the frontal region that lasted for up to 30 minutes at a time and were responsive to therapy with ibuprofen and cold compresses. He did not have aura with his headaches but did have associated photophobia. There was no family history of migraine headaches or other neurological conditions. A review of systems demonstrated no obvious weakness, sensory changes, dysarthria, or dysphagia. He had no systemic symptoms on presentation: cough, rash, joint pains, or night sweats. An optometry exam 6 months prior to admission was normal. He was not taking any medication on a daily basis.

On initial examination he was alert and cooperative. He was afebrile with normal vital signs. His pupils were equal and reactive, his visual fields were normal, and normal visual acuity was documented. Examination of the extraocular movements revealed slight left-sided esotropia. He had dysmetria bilaterally with the finger-to-nose test. He was ataxic, with poor balance, and unable to perform a tandem gait. The rest of the neurological and general examinations were unremarkable. Initial investigations, which included complete blood count, electrolytes, and CT brain scan, were normal.

Over the next 24 hours his clinical status continued to evolve; he developed a severe holocephalic throbbing headache and had dysconjugate eye movements in all directions, followed by a decrease in level of consciousness. On upward gaze, downbeat vertical nystagmus was elicited and horizontal diplopia noted on bidirectional lateral eye movements. Deep tendon reflexes remained normal. Ophthalmological evaluation showed visual acuities of 20/50 OD and 20/30 OS, as well as convergence retraction nystagmus. Fundoscopy was normal. By 48 hours after admission the patient had complete ophthalmoparesis.

A lumbar puncture was completed 48 hours after presentation. Cerebrospinal fluid analysis (CSF) revealed 1 RBC, 0 WBC, glucose 3.0 mmol/L (normal range 2.2–3.9 mmol/L), and protein 0.14 mmol/L (normal range 0.15–0.45 g/L). CSF bacterial culture was negative. CSF viral PCR studies completed for herpes simplex virus, varicella zoster virus, enterovirus, and parechovirus were all negative. Acetylcholine receptor antibody testing was negative. A brain MRI completed 72 hours after presentation showed normal grey and white matter structures of cerebral hemispheres, as well as normal brainstem, cerebellar, and ventricular structures. An electroencephalogram (EEG) was normal for age. Nerve conduction studies (NCS) of the left upper extremity showed normal motor (ulnar nerve) and sensory (median nerve) function. No significant electrodecremental response was seen with repetitive slow frequency (2 Hz) stimulation of the left ulnar nerve during rest and after 1 minute of exercise. The patient was unable to tolerate further testing including more proximal NCS testing (e.g., face) or EMG testing. The GQ1b/GM1 antibody panel was sent and returned negative for antibodies.* Campylobacter jejuni* was isolated from the stool culture and further evidence of a resolving infection was confirmed with serologic markers (positive IgM and IgG).

Following this workup it was felt that BBE was the most likely diagnosis. Due to his complete ophthalmoplegia, treatment with intravenous immunoglobulin (IVIG) for a 5-day treatment period (total dose 2 grams/kg of body weight) was commenced.

After his 5-day treatment course with IVIG, the patient's headaches and level of mentation improved and so he was discharged. At the time of discharge, he had small amplitude vertical gaze bilaterally but still had severe ophthalmoplegia. On follow-up at two weeks, he had progressive improvement of his symptoms; however he had a reoccurrence of diplopia one month after his initial presentation. He had residual deficit of abduction in left gaze and moderately large comitant esotropia (40 prism diopters in near fixation and 45 prism diopters for distance fixation). He was treated with a 5-week tapering dose of prednisone (40 mg initial dose tapering over 5 weeks). During this time, his ocular motility improved significantly. His long-term follow-up needs included prophylaxis therapy with amitriptyline for migraine headaches.

## 3. Discussion

Complete external ophthalmoplegia without ptosis is rarely described in pediatric neurology. The causes of this phenomenon are varied and may involve the neuromuscular junction (e.g., myasthenia gravis), the oculomotor nerves (e.g., MFS, Guillain-Barré syndrome), or the brainstem (BBE, Wernicke's syndrome) [6, 7]. In the context of this patient, other disorders that were considered included viral encephalitis, ophthalmoplegic migraines, and acquired nonaccommodative esotropia of childhood.

Both BBE and MFS have been associated with anti-GQ1b antibodies and* Campylobacter jejuni* gastroenteritis [3–6]. BBE is described in patients presenting with progressive, symmetric ophthalmoplegia and ataxia, as well as a disturbance of consciousness [5, 8]. Patients with MFS have ophthalmoplegia, ataxia, and areflexia [3, 8]. Additionally, patients with these findings and hypersomnolence have BBE [3, 8]. EEG slow wave activity and hyperintense foci on T2 weighted MRI images have been reported in BBE [5]. From 83 to 99% of cases of MFS and Guillain-Barré syndrome with ophthalmoplegia and 68% of BBE show elevated levels of anti-GQ1b antibodies early in the course of illness [6, 8]. The levels of antibodies are typically at their peak when neurological symptoms are most profound and then decrease over time [6]. The exact pathophysiology behind anti-GQ1b antibody syndromes remains unknown; however it is postulated that infectious organisms such as* Campylobacter jejuni* have structurally homologous antigens to human gangliosides which have been found to concentrate in the neuromuscular junction and glial cells [2, 6, 9]. Through molecular mimicry, the cellular immune system identifies both the gangliosides and the infectious agent as foreign antigens. The host immunoglobulins bind to the detected “foreign” antigens resulting in the activation of the membrane attack complex and may lead to injury of nerve terminals and the destruction of Schwann cells [9]. In a case of anti-GQ1b negative MFS or BBE, there may be another antibody against gangliosides that is causing the development of symptoms; however these antibodies have not yet been identified [9].

This case involved a differential diagnosis of myasthenia gravis (less likely from negative acetylcholine receptor antibodies and nonsuggestive NCS), botulism (less likely from negative botulism culture and nonsuggestive NCS), an acute demyelinating syndrome (negative MRI), and MFS, BBE, viral encephalitis, and acquired nonaccommodative esotropia of childhood. Some features were typical of MFS, including the acute onset of ataxia and ophthalmoplegia; however, reflexes were present, and the presence of headache and drowsiness were prominent features suggestive of BBE (however, the MRI and EEG were normal). Acquired nonaccommodative esotropia was less likely based on the responsiveness to therapy.

Differentiating viral encephalitis from BBE in the context of this patient's altered level of consciousness and headaches is crucial due to increased morbidity should the diagnosis of viral encephalitis be missed. In the presence of fever an infective cause should always be considered initially [7]. Poor outcomes of viral encephalitis are associated with diffusion restriction on MRI, presenting with seizures or other focal neurological findings acutely, younger age (<5 years), and infection with herpes simplex virus [10]. Viral encephalitis is a clinical diagnosis based on altered mental status lasting for greater than 24 hours and the presence of a documented fever within 72 hours of presentation, generalized seizures or other new onset focal neurological findings, elevations in the CSF WBC count, and suggestive abnormalities on neuroimaging or electroencephalogram [10]. As BBE is also diagnosed clinically, the history and progression of symptoms are key to differentiating it from viral encephalitis. The absence of fever and lack of disturbance of consciousness on initial presentation in our patient with a history of an antecedent gastrointestinal infection prompted our investigations to take place based on the progression of symptoms. The clinical features of our patient in the context of some supportive serologic markers (*C. jejuni* IgM) are supportive of our diagnostic conclusions but unfortunately are not confirmatory, especially in the absence of a positive anti-GQ1b antibody, as well as a normal EEG/MRI.

There have been other case reports which describe an atypical MFS with patients being anti-GQ1b negative [2, 4, 11]. The presentation of these cases varies, although ataxia and ocular findings were present in all cases (Table 1). None of the previous cases described a recurrence of symptoms, although evidence suggests that recurrence is more likely in younger patients, those with milder illness, and those who are positive for GQ1b antibodies [12]. Other authors have suggested that antibodies against gangliosides other than GQ1b may be playing a role in the pathogenesis of MFS and BBE [13]. Perhaps this case is further evidence that other antibodies against gangliosides still need to be identified.

## 4. Conclusions

This patient represents a case of BBE with negative anti-GQ1b antibodies after a preceding infection with* Campylobacter jejuni*. The recurrence of ocular symptoms that may have had some response to corticosteroid therapy indicated that steroid therapy had no adverse effects although there is no definite benefit in hastening recovery. It may be considered as a possible therapy for similar patients exhibiting symptoms of relapse. Evidence of successful steroid treatment for symptom recurrence was shown in one anti-GQ1b antibody positive adult case report of MFS, but evidence is extremely limited and there are no pediatric reports to the best of our knowledge [14]. Treatment with corticosteroids for acute postinfectious inflammatory syndromes such as Guillain-Barré syndrome and MFS remains controversial [12, 15].

## Figures and Tables

**Table 1:** Prior treatments for anti-GQ1b negative cases and the associated symptoms.

Author	Diagnosis	Primary symptoms	Therapy	Outcome
Tan et al., 2003 [2]	Miller Fisher syndrome with negative anti-GQ1b immunoglobulin G antibodies	Ataxia Areflexia Bilateral ptosis Cranial nerve III and VI palsies	Intravenous immunoglobulin (400 mg/kg/day) for 5 days	External ocular movement improvement within 2 weeks and complete resolution within 1 month

Akinci et al., 2010 [11]	Anti-GQ1b negative Miller Fisher syndrome (after suspected *Mycoplasma pneumoniae* infection)	Ataxia Areflexia Left-sided horizontal gaze palsy Bulbar palsy	Intravenous immunoglobulin (400 mg/kg/day) for 5 days	Her left-sided gaze palsy improved within 2 weeks, as well as ataxia, dysarthria, and dysphagia; after 4 weeks there were no residual deficits

Lee, 2012 [4]	Anti-GQ1b negative Miller Fisher syndrome after *Campylobacter jejuni *infection	Ataxia Areflexia Left-sided ptosis	Intravenous immunoglobulin (1 g/kg/day) for 2 days	The patient was able to walk 7 days after therapy and had improvement in ptosis by posttreatment day 11; after 2 months only minor intermittent diplopia remained
